# Nrf2/BAX/Bcl2 Signaling Crosstalk and Histopathological Correlates of Renoprotection by Black Soldier Fly Larval Oil Against Aflatoxin B1-Induced Oxidative and Inflammatory Kidney Injury in Rats

**DOI:** 10.5812/ijpr-173736

**Published:** 2026-08-24

**Authors:** Yalda Arast, Fateme Heidari

**Affiliations:** 1 Research Center for Environmental Pollutants, Qom University of Medical Sciences Qom Iran; 2 Department of Anatomical Sciences, School of Medicine, Qom University of Medical Sciences Qom Iran

**Keywords:** Aflatoxin B1, *Hermetia illucens*, NF-E2-Related Factor 2, Bcl-2-Associated X Protein, Proto-Oncogene Proteins c-Bcl-2, Nephrotoxicity, Oxidative Stress, Inflammation, Histopathology, Apoptosis

## Abstract

**Background:**

Aflatoxin B1 (AFB1) is a highly hazardous mycotoxin commonly detected in contaminated food supplies and poses serious health risks to both humans and animals. Although its hepatotoxic effects are well established, the kidney, a principal organ for toxin elimination, remains highly susceptible to AFB1-induced injury through mechanisms involving oxidative stress, inflammatory responses, and apoptotic cell death. Black soldier fly larvae (BSFL) oil, characterized by a high content of medium-chain fatty acids and other bioactive lipids, represents a sustainable and promising source of naturally derived antioxidant and anti-inflammatory agents.

**Objectives:**

This study aimed to evaluate the renoprotective potential of the n-hexane BSFL extract against AFB1-induced nephrotoxicity in a rat model, with particular emphasis on elucidating the involvement of Nrf2-driven antioxidant pathways and BAX/Bcl2-regulated apoptotic signaling.

**Methods:**

Thirty-five male Wistar rats were allocated to five experimental groups: control, AFB1 (75 µg/kg), BSFL (360 mg/kg), AFB1 + BSFL180, and AFB1 + BSFL360. After a 28-day treatment period, renal function indicators (BUN, creatinine, uric acid), oxidative stress markers (MDA, SOD, CAT, GSH), inflammatory cytokines (TNF-α, IL-6, NF-κB), and apoptosis-related proteins (Nrf2, BAX, Bcl2) were evaluated. Histopathological assessments were also performed to examine tubular necrosis, glomerular damage, and inflammatory infiltration.

**Results:**

AFB1 exposure resulted in marked increases in BUN, creatinine, uric acid, MDA, TNF-α, IL-6, NF-κB, and BAX levels, along with significant decreases in SOD, CAT, GSH, Nrf2, and Bcl2 (P < 0.001). Co-administration of BSFL extract, particularly at the higher dose (360 mg/kg), effectively reversed these alterations and restored the BAX/Bcl2 ratio toward normal values. Histological examination corroborated these biochemical improvements by showing reduced tubular necrosis, glomerular injury, and inflammatory cell infiltration in the extract-treated groups.

**Conclusions:**

These findings indicate that BSFL extract confers substantial renoprotection against AFB1-induced kidney injury through the coordinated regulation of Nrf2-mediated antioxidant defense and BAX/Bcl2-dependent apoptotic pathways. The absence of intrinsic toxicity, together with the sustainable and eco-friendly production of BSFL oil, underscores its potential as a promising natural therapeutic candidate for mitigating mycotoxin-associated nephrotoxicity.

## 1. Background

Mycotoxins are secondary metabolites produced by filamentous fungi and represent one of the most significant categories of naturally occurring food contaminants. Among these, aflatoxins—particularly aflatoxin B1 (AFB1)—are exceptionally hazardous because of their widespread presence in staple agricultural products, including cereals, oilseeds, spices, and tree nuts (1). AFB1 is notoriously resistant to heat and conventional food-processing methods, which amplifies its public health impact (2). Once ingested, this mycotoxin is metabolized in the liver via cytochrome P450 enzymes, yielding reactive intermediates that initiate a cascade of cellular damage (3). Although the hepatotoxic profile of AFB1 has been thoroughly investigated, emerging evidence indicates that the kidney, a major excretory organ, is also profoundly affected by AFB1, a facet that remains underexplored (4).

Three interconnected processes drive AFB1-related kidney damage: unchecked generation of reactive oxygen species (ROS), sustained activation of inflammatory pathways, and dysregulated cell-death programs (5). When ROS output outpaces the cell’s antioxidant capacity—a defense network built around enzymes such as superoxide dismutase (SOD) and catalase (CAT), together with the nonenzymatic scavenger glutathione (GSH)—the resulting redox imbalance peroxidizes membrane lipids, disrupts mitochondrial function, and ultimately injures tubular epithelial cells (6). In parallel, AFB1 provokes the release of inflammatory cytokines such as tumor necrosis factor-alpha (TNF-α), interleukin-1β (IL-1β), and interleukin-6 (IL-6), which recruit immune cells and exacerbate tissue destruction (7). At the molecular level, the transcription factor Nrf2 normally drives the expression of a battery of cytoprotective antioxidant genes; when AFB1 suppresses this pathway, renal defenses against oxidative injury are weakened. An additional regulatory layer involves the ratio between the pro-apoptotic protein BAX and its anti-apoptotic partner Bcl-2, which sets the threshold for mitochondria-mediated cell death in renal tissue and is commonly disrupted in toxin-induced nephropathy (8). More recent work has also linked AFB1 nephrotoxicity to defective mitophagy and mitochondrial pore opening, pointing to a shared molecular thread connecting pyroptosis and apoptosis (9).

Alongside mounting concern over mycotoxin contamination, attention has shifted toward sustainable, environmentally friendly sources of bioactive molecules with therapeutic promise. Larvae of the black soldier fly (*Hermetia illucens*) are notable in this regard for their capacity to convert organic waste streams into nutrient-dense biomass (10). Oil recovered from the larvae by n-hexane extraction is dominated by medium-chain fatty acids; in the batch used here, lauric acid alone accounted for 48% of the total fatty acid content. The same oil also contains tocopherols, sterols, and phospholipids, which together confer antioxidant and anti-inflammatory properties (11). Earlier work has shown that this larval oil can attenuate oxidative injury and inflammatory cascades across several models of chemically induced toxicity, apparently by engaging both the Nrf2 and NF-κB pathways (12). Beyond its biological activity, rearing black soldier fly larvae aligns with circular-economy principles by converting low-value organic residues into valuable bioactive material without the land-use or deforestation pressures associated with conventional oil crops (13, 14).

Yet, despite this accumulating evidence of bioactivity, no study has systematically tested whether BSFL oil can protect the kidney against AFB1, nor has it been examined whether such protection, if present, would operate through the Nrf2 antioxidant axis or the BAX/Bcl-2 apoptotic balance.

## 2. Objectives

Because the kidney is particularly susceptible to AFB1-driven oxidative and inflammatory injury, and because BSFL larval oil is known to possess antioxidant capacity, we hypothesized that it would meaningfully limit AFB1 nephrotoxicity. Therefore, this study aimed, for the first time, to determine whether an n-hexane BSFL extract can protect rat kidneys from AFB1 injury by jointly assessing renal function, oxidative stress markers, inflammatory cytokines, histopathology, and the coordinated behavior of the Nrf2 and BAX/Bcl-2 pathways.

## 3. Methods

### 3.1. Reagents and Chemicals

Aflatoxin B1 (AFB1) (≥ 98% purity) and n-hexane were obtained from Sigma-Aldrich (USA). Corn oil was used as the vehicle for all oral administrations. Reagents for ELISA-based quantification of renal function biomarkers (BUN, creatinine, uric acid), oxidative stress indicators (SOD, CAT, GSH, MDA), inflammatory markers (TNF-α, IL-6, IL-1β), and apoptosis-regulatory proteins (Nrf2, Bcl-2, Bax) were purchased from Sunred (China) and other accredited vendors.

### 3.2. Preparation of Black Soldier Fly Larvae n-Hexane Extract

Larval material was obtained from a colony maintained at Qom University of Medical Sciences, where the insects were fed a substrate of fruit and olive processing residues under controlled conditions (27 ± 2 °C; relative humidity 65% - 70%). Prepupae were harvested by hand-picking, rinsed, and dehydrated in an oven at 60 °C for 24 hours. The dried larvae were then ground into a fine powder. Lipid extraction was performed using a Soxhlet apparatus with n-hexane as the solvent, with a 3-hour extraction cycle. After solvent removal by rotary evaporation under reduced pressure, the resulting oil fraction was stored at 20 °C in a nitrogen-purged environment until use. The extraction yield was within the typical range reported for n-hexane extraction of black soldier fly larvae oil (11, 12). All animal experiments were conducted using a single batch of extract to ensure consistency; the fatty acid profile reported in section 3.3 was determined using an aliquot from the same batch. For each administration, an aliquot of the stored extract was thawed and dissolved in corn oil immediately before dosing (10).

### 3.3. Fatty Acid Profiling of BSFL Extract Using GC-MS

After derivatization to fatty acid methyl esters, samples were analyzed by GC-MS at an accredited IFDA facility. Compounds were identified by comparing mass spectra with the NIST reference library. Lauric acid (C12:0) was the predominant fatty acid, comprising 48% of the total lipid profile, whereas other fatty acids were present in minor proportions.

### 3.4. Animals

Male Wistar rats (n = 35) were used in this study. Baseline body weight ranged from 200 to 250 g, and animals were 10 to 12 weeks old. Housing conditions were maintained throughout the study: ambient temperature 22 ± 2 °C, humidity 50% to 60%, and a 12-hour light/12-hour dark cycle. Standard laboratory chow and water were provided ad libitum. All animal procedures were approved by the relevant ethics committee under clearance code IR.MUQ.AEC.1403.006.

### 3.5. Experimental Design and Dose Selection

After a one-week acclimatization period, animals were randomly allocated to five experimental groups (n = 7 per group) and treated for 28 days as follows:

Control group: received corn oil vehicle (2 mL/kg body weight) daily.

AFB1 group: received AFB1 alone at 75 µg/kg body weight (4).

AFB1 + BSFL180 group: received AFB1 (75 µg/kg) and BSFL extract at the lower dose (180 mg/kg) concurrently (co-administered) daily.

AFB1 + BSFL360 group: received AFB1 (75 µg/kg) and BSFL extract at the higher dose (360 mg/kg) concurrently (co-administered) daily.

BSFL360 group: received BSFL extract alone at 360 mg/kg, without AFB1 exposure.

Accordingly, BSFL extract was administered concurrently with AFB1 (co-exposure model) for 28 consecutive days, rather than as a pretreatment or as a therapeutic intervention after injury establishment (4, 6). This design evaluated the protective effect of the extract against the development of AFB1-induced nephrotoxicity, rather than its ability to reverse established injury. On day 29, approximately 24 hours after the final gavage, all animals were euthanized, and samples were collected.

The rationale for selecting BSFL extract doses of 180 and 360 mg/kg was based on the extract fatty acid composition, particularly its lauric acid content (48% of total fatty acids), as confirmed by GC-MS. These doses were calculated to provide approximately 86 and 173 mg/kg/day of lauric acid, respectively, corresponding to the protective dose window previously reported for pure lauric acid in rodent injury models. The AFB1 dose (75 µg/kg) was selected based on prior studies demonstrating reproducible renal oxidative injury without systemic lethality (4). The 28-day treatment period was selected in accordance with established nephrotoxicity models and to allow sufficient time for the development of oxidative stress and inflammatory responses (4, 6).

All treatments were administered at a fixed time in the morning (9:00 - 10:00 AM) to minimize circadian variability. Body weight was recorded at baseline, weekly thereafter, and again on day 29 immediately before euthanasia.

### 3.6. Biological Sample Harvesting

Approximately 24 hours after the final gavage (day 29), each rat was reweighed and anesthetized by intraperitoneal injection of ketamine (50 mg/kg) and xylazine (10 mg/kg) (15). Adequate anesthesia was confirmed by the absence of a withdrawal response to toe pinch. Blood was collected by cardiac puncture using sterile syringes and transferred into plain tubes (without anticoagulant) for serum separation. Samples were allowed to clot at room temperature for 30 minutes and then centrifuged at 3000 rpm for 15 minutes at 4 °C. The serum supernatant was carefully collected, aliquoted, and stored at −20 °C until biochemical analysis.

Immediately after blood collection, rats were euthanized by cervical dislocation under deep anesthesia to ensure a painless procedure. Both kidneys were rapidly excised, cleared of adherent fat and connective tissue, and weighed individually. The right kidney was snap-frozen in liquid nitrogen and then stored at −80 °C for subsequent biochemical and molecular analyses. The left kidney was fixed in 10% neutral-buffered formalin for histopathological processing.

### 3.7. Renal Function Evaluation

Serum BUN, creatinine, and uric acid concentrations were measured using colorimetric assay kits (Pars Azmoon Inc., Tehran, Iran) according to the manufacturer’s instructions. Absorbance was measured using a spectrophotometer (Unico 1200, USA) at the appropriate wavelength for each analyte, and concentrations (expressed as mg/dL) were calculated using concurrently generated standard curves.

### 3.8. Oxidative Stress Profiling in Kidney Homogenates

Frozen kidney tissue was homogenized in phosphate buffer (0.1 M, pH 7.4) and centrifuged (10,000 × g, 15 minutes, and 4 °C). The supernatants were used for protein determination (Bradford method) (16) and for oxidative stress assays using commercial kits (Navand Lab, Iran):

- SOD: NBT reduction inhibition, U/mg protein (17)

- CAT: H_2O_2 decomposition at 240 nm, U/mg protein (18)

- GSH: Ellman's reaction at 412 nm, μmol/g tissue (19)

- MDA: TBA reaction at 532 nm, nmol/mg protein (20)

### 3.9. Inflammatory Cytokine Quantification

The same kidney homogenate supernatants were used to quantify pro-inflammatory cytokines using rat-specific commercial ELISA kits (Karmania Pars Gene Inc., Tehran, Iran) for TNF-α, IL-6, and NF-κB, following the method described by Van Weemen and Schuurs (21). Cytokine levels were normalized to total protein content and expressed as pg/mg protein.

### 3.10. Protein Expression Analysis via Western Blotting

Total protein was extracted from kidney tissue using RIPA lysis buffer. Equal amounts of protein (20 μg per lane) were separated by SDS-PAGE and transferred to PVDF membranes. Membranes were blocked with BSA to prevent nonspecific antibody binding. Primary antibodies against Nrf2, Bcl-2, Bax, and β-actin (loading control) were incubated overnight at 4 °C at dilutions of 1:1000 for target proteins and 1:2500 for β-actin. After several washes with TBST, membranes were incubated with an HRP-conjugated secondary antibody (1:10000 dilution), and bands were visualized using ECL chemiluminescence. Band intensities were quantified by densitometry using ImageJ (NIH) (22).

### 3.11. Histological Assessment

Formalin-fixed tissues were processed into paraffin blocks, sectioned at 4 - 5 μm, and stained with hematoxylin and eosin (H&E) (23) and Masson's trichrome for collagen visualization. For each animal, 10 randomly selected, non-overlapping microscopic fields (× 400 magnification) from three non-consecutive sections were evaluated by a pathologist blinded to group allocation. Tubular necrosis was calculated as the proportion of necrotic tubules (defined by loss of epithelial cell nuclei, cytoplasmic vacuolation, and cellular debris) relative to the total tubules counted per field. Necrotic glomeruli were expressed as the percentage of glomeruli exhibiting capillary loop collapse, mesangial cell lysis, or nuclear pyknosis relative to the total glomeruli counted. Inflammatory infiltration was scored as the percentage of the field area occupied by inflammatory cells. Lesion severity for H&E parameters was also graded on a 0 - 3 scale by a pathologist blinded to group assignments (23).

### 3.12. Statistical Analysis

Statistical analyses were performed using GraphPad Prism software (version 9.0). Quantitative data are presented as mean ± standard deviation (SD). Normality was assessed using the Shapiro-Wilk test, and homogeneity of variances was evaluated using Levene’s test. For normally distributed data (biochemical parameters, oxidative stress markers, inflammatory cytokines, and Western blot densitometry), group differences were analyzed by one-way ANOVA followed by Tukey's post hoc test. For non-parametric data (histopathological scores), the Kruskal-Wallis test followed by Mann-Whitney U tests with Bonferroni correction was used. The statistical unit for all analyses was the individual animal (n = 7 per group). A P value below 0.05 was considered statistically significant.

### 3.13. Ethical Considerations

All animal procedures adhered to the NIH Guide for the Care and Use of Laboratory Animals, and the protocol was approved by the Institutional Animal Ethics Committee of Qom University of Medical Sciences (Approval Code: IR.MUQ.AEC.1403.006). Kidney specimens were obtained from a previously approved study of aflatoxin-induced liver injury and, under the same approval, were additionally used for renal molecular and histopathological analyses in accordance with the 3Rs principles of replacement, reduction, and refinement. Every effort was made to minimize animal suffering and to keep group sizes to the minimum required for statistically meaningful results. Animals were euthanized by cervical dislocation under deep anesthesia, in accordance with the 2020 AVMA Guidelines for the Euthanasia of Animals and with the committee's explicit approval.

## 4. Results

### 4.1. Effects of BSFL Extract on Renal Function Biomarkers

**Figure 1.**
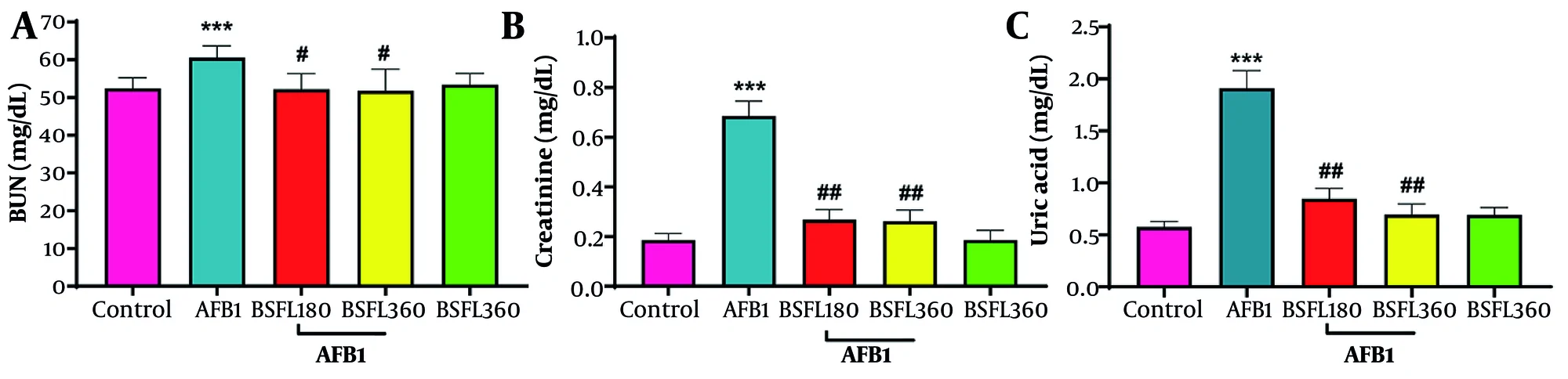
Effects of n-hexane BSFL extract on renal function biomarkers in AFB1-induced nephrotoxic rats. (A) Serum blood urea nitrogen (BUN) levels; (B) Serum creatinine concentrations; (C) Serum uric acid levels. Data are expressed as mean ± SD (n = 7 per group). ***P < 0.001 compared with the control group; #P < 0.05, ##P < 0.01, and ###P < 0.001 compared with the AFB1-treated group (one-way ANOVA followed by Tukey's post hoc test).

The effects of AFB1 exposure and subsequent co-administration of BSFL extract on serum markers of renal function, namely BUN, creatinine, and uric acid, are shown in Figure 1A–C.

As shown in Figure 1A, rats exposed to AFB1 alone exhibited a marked elevation in BUN concentrations (59.5 mg/dL) relative to the control group (51.0 mg/dL). This increase was substantially attenuated in animals receiving either dose of the BSFL extract, with both the 180 mg/kg and 360 mg/kg doses restoring BUN levels to 51.0 mg/dL; these values were statistically indistinguishable from those of the control group. Notably, the group receiving BSFL extract alone (360 mg/kg) exhibited a BUN level of 51.5 mg/dL, confirming the absence of intrinsic nephrotoxicity.

Similarly, creatinine levels (Figure 1B) followed an analogous pattern. AFB1 intoxication caused a significant rise in serum creatinine (0.69 mg/dL) compared with the control group (0.18 mg/dL). Co-treatment with BSFL extract effectively reversed this elevation in a dose-dependent manner, with the 180 mg/kg dose reducing creatinine to 0.26 mg/dL and the 360 mg/kg dose further lowering it to 0.25 mg/dL. The extract-alone group displayed a creatinine level (0.18 mg/dL) comparable to that of the healthy controls.

Uric acid concentrations (Figure 1C) were also adversely affected by AFB1 exposure, showing a substantial increase (1.95 mg/dL) relative to control values (0.58 mg/dL). Administration of the BSFL extract alongside the toxin significantly mitigated this rise, with the 180 mg/kg dose reducing uric acid to 0.85 mg/dL and the 360 mg/kg dose further decreasing it to 0.75 mg/dL. The extract-alone group exhibited a uric acid level of 0.75 mg/dL, which was comparable to that of the control group.

### 4.2. Effects of BSFL Extract on Renal Oxidative Stress Biomarkers

**Figure 2.**
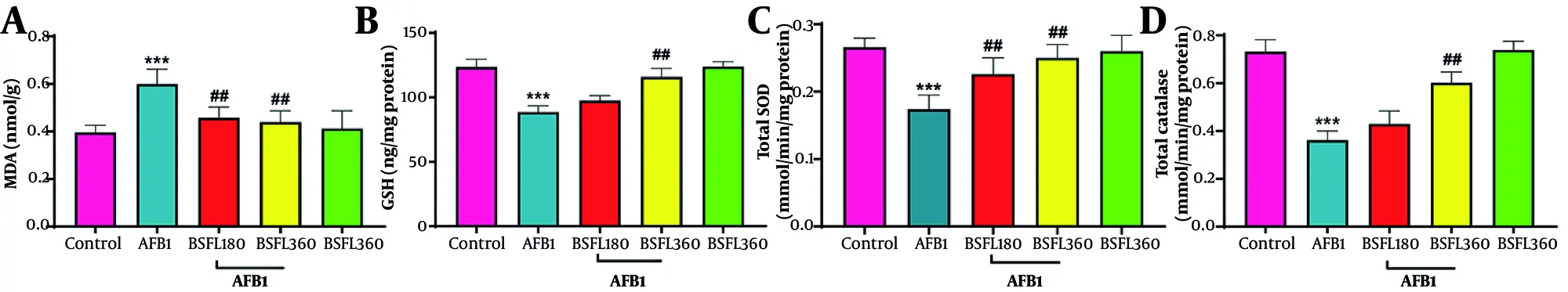
Effects of n-hexane BSFL extract on renal oxidative stress parameters in AFB1-induced nephrotoxic rats. (A) Malondialdehyde (MDA) levels as an index of lipid peroxidation; (B) Reduced glutathione (GSH) content; (C) Superoxide dismutase (SOD) activity; (D) Catalase (CAT) activity. Data are expressed as mean ± SD (n = 7 per group). ***P < 0.001 compared with the control group; #P < 0.05 and ##P < 0.01 compared with the AFB1-treated group (one-way ANOVA followed by Tukey's post hoc test).

The effects of BSFL extract on oxidative stress parameters are shown in Figure 2A–D.

#### 4.2.1. Lipid Peroxidation (MDA)

Figure 2A illustrates changes in malondialdehyde (MDA) content, a reliable indicator of lipid peroxidation. AFB1 treatment provoked a marked increase in renal MDA levels (0.59 mmol/g) relative to the control group (0.39 mmol/g). Co-administration of BSFL extract at 180 mg/kg reduced MDA to 0.46 mmol/g, whereas the 360 mg/kg dose produced a more robust decline to 0.44 mmol/g, bringing values closer to the normal range. The extract-alone group exhibited MDA levels of 0.41 mmol/g, comparable to those of the control group, confirming the absence of pro-oxidant activity.

#### 4.2.2. Reduced Glutathione (GSH) Content

Figure 2B depicts changes in renal GSH content, a critical non-enzymatic antioxidant. AFB1 administration caused marked depletion of GSH (88 ng/mg protein) compared with the control group (125 ng/mg protein). Treatment with BSFL extract at 180 mg/kg increased GSH to 95 ng/mg protein, while the higher dose (360 mg/kg) further increased it to 110 ng/mg protein. The extract-alone group displayed GSH levels of 120 ng/mg protein, which were statistically indistinguishable from the control values, confirming the safety of the extract.

#### 4.2.3. Superoxide Dismutase (SOD) Activity

SOD activity, a key enzymatic antioxidant, is presented in Figure 2C. AFB1 exposure resulted in a significant reduction in SOD activity (0.18 mmol/min/g protein) relative to the control group (0.26 mmol/min/g protein). Co-treatment with BSFL extract at 180 mg/kg increased SOD activity to 0.23 mmol/min/g protein, while the 360 mg/kg dose further increased it to 0.24 mmol/min/g protein. The extract-alone group showed SOD activity of 0.25 mmol/min/g protein, comparable to that of the healthy controls.

#### 4.2.4. Catalase (CAT) Activity

Figure 2D summarizes changes in renal catalase activity. AFB1 intoxication provoked a pronounced decrease in CAT activity (0.36 mmol/min/g protein) compared with the control group (0.74 mmol/min/g protein). Treatment with BSFL extract at 180 mg/kg partially restored CAT activity to 0.42 mmol/min/g protein, while the higher dose (360 mg/kg) produced a more substantial recovery to 0.59 mmol/min/g protein. The extract-alone group exhibited CAT activity of 0.72 mmol/min/g protein, which was nearly identical to the control value.

### 4.3. Effects of BSFL Extract on Renal Inflammatory Markers

**Figure 3.**
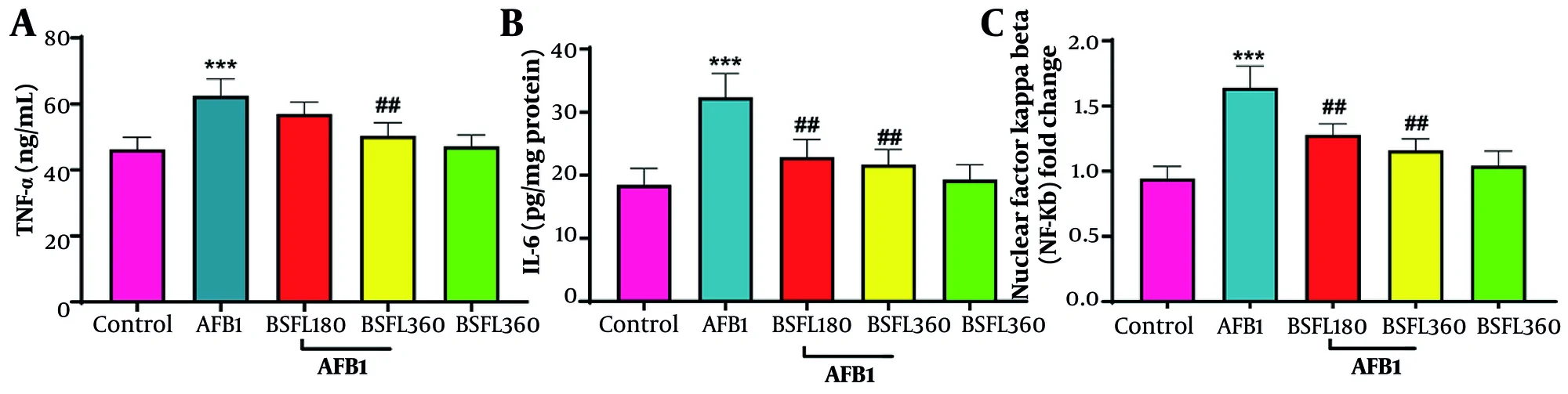
Effects of n-hexane BSFL extract on renal inflammatory markers in AFB1-induced nephrotoxic rats. (A) Tumor necrosis factor-alpha (TNF-α) levels; (B) Interleukin-6 (IL-6) concentrations; (C) Nuclear factor kappa-B (NF-κB) protein expression (fold change relative to control). Data are expressed as mean ± SD (n = 7 per group). ***P < 0.001 compared with the control group; #P < 0.05, ##P < 0.01, and ###P < 0.001 compared with the AFB1-treated group (one-way ANOVA followed by Tukey's post hoc test).

The levels of the pro-inflammatory mediators TNF-α, IL-6, and NF-κB were evaluated in kidney tissues, and the results are presented in Figure 3A–C.

As depicted in Figure 3A, AFB1 challenge elicited a substantial elevation in TNF-α concentrations (62 pg/mL) relative to the control group (46 pg/mL). This response was effectively suppressed by concurrent BSFL extract administration, with the 180 mg/kg dose reducing TNF-α to 57 pg/mL and the 360 mg/kg dose further decreasing it to 50 pg/mL. The extract-alone group exhibited TNF-α levels of 46 pg/mL, identical to those of the control group, confirming the lack of pro-inflammatory activity of the extract.

IL-6 levels (Figure 3B) were also markedly elevated following AFB1 exposure (32.0 pg/mg protein) compared with the control group (18.0 pg/mg protein). Treatment with BSFL extract substantially lowered these values, with the 180 mg/kg dose reducing IL-6 to 22.0 pg/mg protein and the 360 mg/kg dose further decreasing it to 21.0 pg/mg protein. The extract-alone group displayed IL-6 levels of 19.5 pg/mg protein, which were comparable to those of the healthy controls.

NF-κB protein expression, a master transcription factor governing inflammatory responses, is shown in Figure 3C. AFB1 administration caused marked upregulation of NF-κB (1.65-fold change) compared with the control group (0.95-fold change). Co-treatment with the BSFL extract dose-dependently reduced NF-κB expression, with the 180 mg/kg dose lowering it to 1.30-fold and the 360 mg/kg dose further decreasing it to 1.15-fold. The extract-alone group exhibited NF-κB expression of 1.05-fold, which was comparable to the control value.

### 4.4. Effects of BSFL Extract on Apoptosis-Related Protein Expression

**Figure 4.**
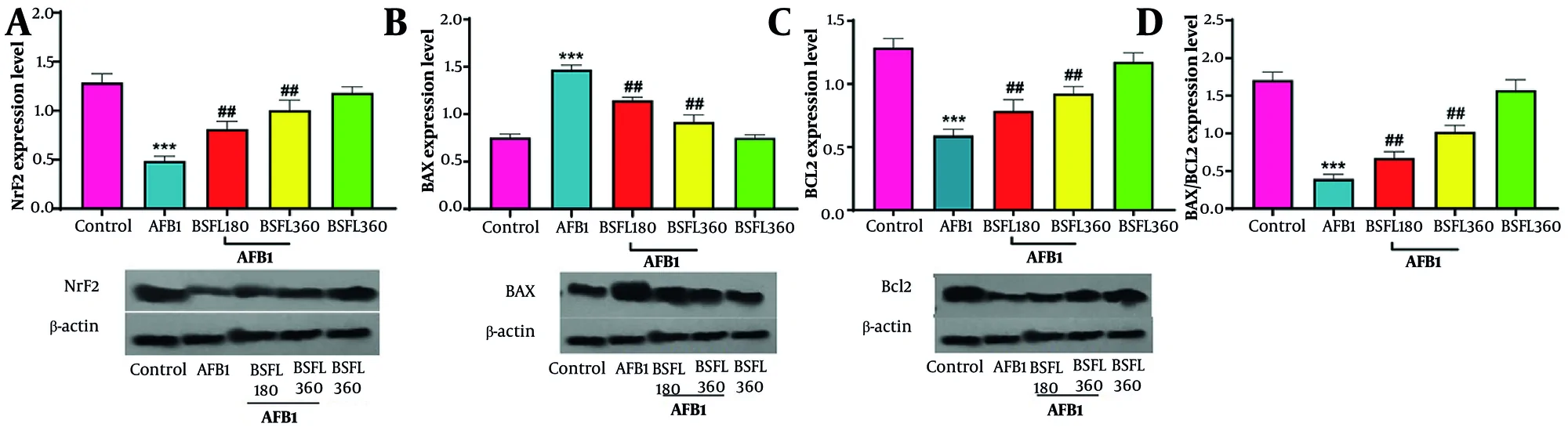
Effects of n-hexane BSFL extract on apoptosis-related protein expression in renal tissues of AFB1-induced nephrotoxic rats. (A) Nrf2 protein expression (fold change relative to control); (B) BAX protein expression (fold change relative to control); (C) Bcl-2 protein expression (fold change relative to control); (D) BAX/Bcl-2 ratio. Protein levels were determined by Western blotting and normalized to β-actin. Data are expressed as mean ± SD (n = 7 per group). ***P < 0.001 compared with the control group; #P < 0.05, ##P < 0.01, and ###P < 0.001 compared with the AFB1-treated group (one-way ANOVA followed by Tukey's post hoc test).

Western blot analysis was performed to assess the protein expression levels of Nrf2, Bcl2, and BAX in renal tissues. The corresponding densitometric quantifications are presented in Figure 4A–D.

As illustrated in Figure 4A, AFB1 exposure caused significant downregulation of Nrf2 protein levels (0.45-fold) compared with the control group (1.30-fold). Co-treatment with the BSFL extract dose-dependently restored Nrf2 expression, with the 180 mg/kg dose increasing it to 0.85-fold and the 360 mg/kg dose further increasing it to 1.00-fold. The extract-alone group exhibited Nrf2 expression of 1.15-fold, which was comparable to the control value.

The expression of the pro-apoptotic protein BAX is shown in Figure 4B. AFB1 administration provoked a marked increase in BAX levels (1.45-fold) relative to the control group (0.75-fold). Treatment with the BSFL extract significantly reduced BAX expression in a dose-dependent manner, with the 180 mg/kg dose lowering it to 1.15-fold and the 360 mg/kg dose further decreasing it to 0.90-fold. The extract-alone group showed BAX expression of 0.80-fold, which was comparable to the control value.

As presented in Figure 4C, the anti-apoptotic protein Bcl2 was markedly reduced following AFB1 administration (0.60-fold) compared with the control group (1.30-fold). Co-treatment with the BSFL extract effectively reversed this alteration, with the 180 mg/kg dose increasing Bcl2 to 0.80-fold and the 360 mg/kg dose further increasing it to 1.00-fold. The extract-alone group exhibited Bcl2 expression of 1.20-fold, which was statistically indistinguishable from the control value.

The calculated BAX/Bcl2 ratio, a critical determinant of the mitochondrial apoptotic pathway, is shown in Figure 4D. AFB1 intoxication resulted in a substantial increase in this ratio (1.7-fold) compared with the control group (0.4-fold), indicating a shift toward apoptotic cell death. Co-administration of the BSFL extract significantly reduced the BAX/Bcl2 ratio, with the 180 mg/kg dose lowering it to 0.65-fold and the 360 mg/kg dose producing a more marked correction to 1.05-fold. The extract-alone group exhibited a ratio of 1.6-fold, which was comparable to the control value.

### 4.5. Histopathological Findings in Kidney Tissues

**Figure 5.**
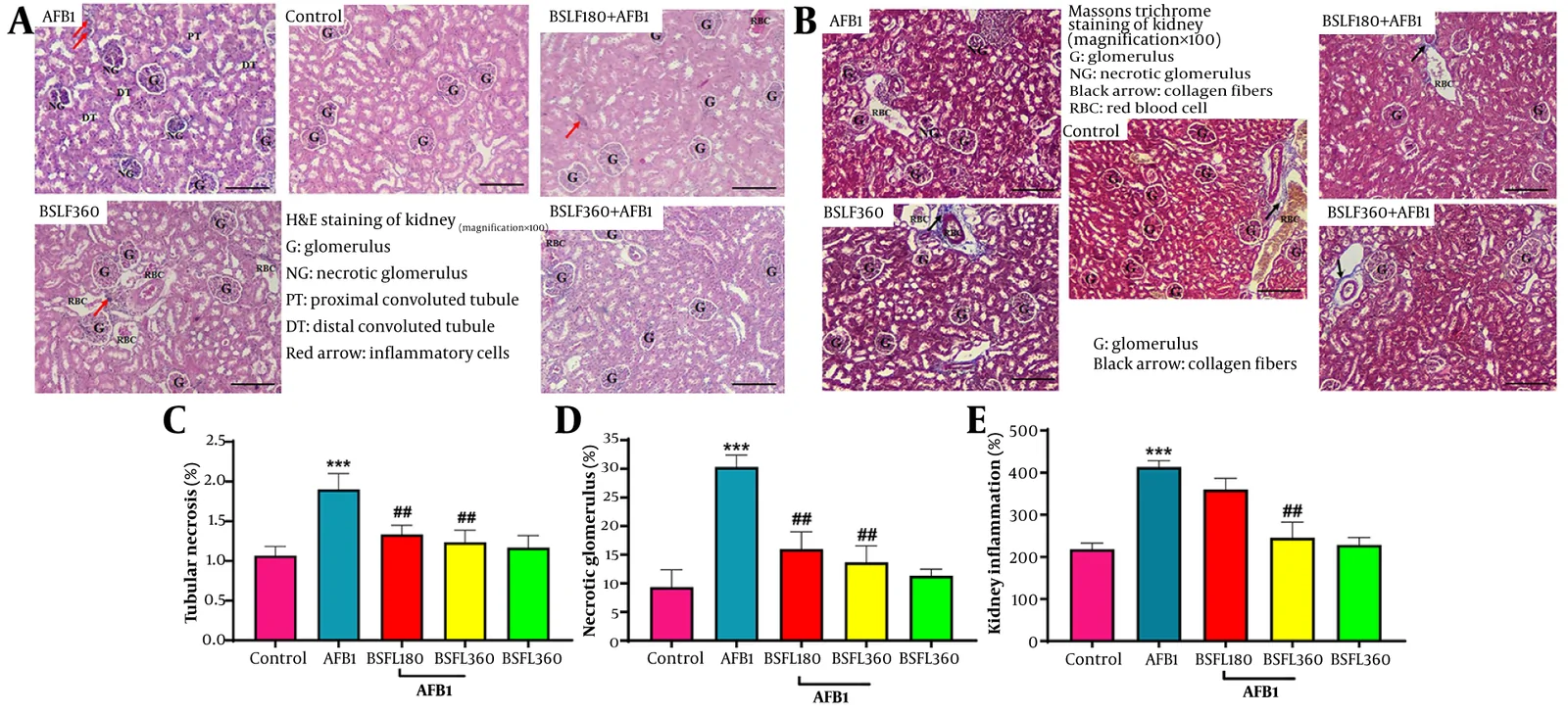
(A) Representative photomicrographs of hematoxylin and eosin (H&E)-stained kidney sections (magnification × 100) from experimental groups. Control (Vehicle): normal renal architecture with intact glomeruli (G), proximal (PT) and distal (DT) convoluted tubules, and no inflammatory infiltration. AFB1: severe renal injury characterized by extensive tubular necrosis, necrotic glomeruli (NG), interstitial inflammatory cell infiltration (red arrows), and red blood cell (RBC) extravasation. AFB1 + BSFL180: moderate improvement with reduced necrosis and diminished inflammatory cells, although some residual abnormalities remain. AFB1 + BSFL360: significant restoration of renal structure with near-normal glomeruli, minimal necrosis, and sparse inflammatory infiltration. BSFL360 alone: normal architecture comparable to control, confirming non-toxicity of the extract. (B) Representative photomicrographs of Masson's trichrome-stained kidney sections (magnification × 100) demonstrating collagen fiber deposition (black arrows). Control (Vehicle): minimal collagen confined to perivascular regions. AFB1: marked interstitial and periglomerular collagen accumulation indicating fibrosis. AFB1 + BSFL180: partial reduction in collagen deposition. AFB1 + BSFL360: pronounced decrease in collagen fibers with near-normal interstitial architecture. BSFL360 alone: collagen pattern similar to control. (C) Quantitative analysis of tubular necrosis (%). (D) Quantitative analysis of necrotic glomeruli (%). (E) Quantitative analysis of interstitial inflammatory infiltration (%). Data are expressed as mean ± SD (n = 7 per group). ***P < 0.001 compared with the control group; #P < 0.05, ##P < 0.01, and ###P < 0.001 compared with the AFB1-treated group (Kruskal-Wallis test followed by Mann-Whitney U test). G: glomerulus; NG: necrotic glomerulus; PT: proximal convoluted tubule; DT: distal convoluted tubule; RBC: red blood cell.

The protective efficacy of BSFL extract against AFB1-induced renal injury was further substantiated by histopathological examination of kidney sections stained with hematoxylin and eosin (H&E) and Masson's trichrome. Representative photomicrographs from each experimental group are presented in Figure 5A–B, and quantitative analyses of histopathological alterations, including tubular necrosis, necrotic glomeruli, and interstitial inflammatory infiltration, are summarized in Figure 5C–E.

#### 4.5.1. H&E Staining Findings

The control group (vehicle) exhibited normal renal histoarchitecture, characterized by intact glomeruli (G) with well-defined Bowman's spaces, distinct proximal (PT) and distal (DT) convoluted tubules with preserved epithelial lining, and no evidence of inflammatory cell infiltration, interstitial edema, or hemorrhage Figure 5A.

In contrast, the AFB1-treated group displayed severe renal damage, including extensive tubular necrosis, pronounced tubular dilatation, and numerous necrotic glomeruli (NG). Prominent interstitial inflammatory cell infiltration (indicated by red arrows) and red blood cell (RBC) extravasation were observed, indicating marked tissue injury and hemorrhage.

The BSFL360 alone group exhibited normal renal architecture comparable to that of the control group, with intact glomeruli, well-preserved tubular structures, and no discernible pathological changes, confirming the non-toxic profile of the extract at the tested dose.

Co-administration of the lower dose of BSFL extract (AFB1 + BSFL180) resulted in noticeable histopathological improvement. The number of necrotic glomeruli was reduced, tubular injury was less severe, and inflammatory infiltration was diminished compared with that in the AFB1 group. However, some residual abnormalities, including mild tubular dilatation and occasional inflammatory cells, remained.

The higher dose of BSFL extract (AFB1 + BSFL360) afforded marked protection, with renal sections showing near-complete restoration of normal architecture. Glomeruli appeared largely intact, tubular necrosis and dilatation were markedly attenuated, and inflammatory cell infiltration was significantly reduced. Renal tissue in this group closely resembled that of the healthy control animals.

#### 4.5.2. Masson's Trichrome Staining Findings

The control group (vehicle) displayed minimal collagen deposition, with only a few thin collagen fibers (black arrows) confined to the perivascular and periglomerular regions, indicating normal renal architecture Figure 5B.

The AFB1-treated group exhibited a marked increase in collagen fiber deposition within the renal interstitium and around the glomeruli, confirming the development of interstitial fibrosis. This was accompanied by necrotic glomeruli and red blood cell accumulation, further confirming severe renal injury.

The BSFL360 alone group displayed a collagen deposition pattern similar to that of the control group, with minimal interstitial fibrosis and no evidence of pathological collagen accumulation.

The AFB1 + BSFL180 group showed a partial reduction in collagen fiber deposition compared with that in the AFB1 group. Although some fibrotic areas persisted, the overall extent of interstitial fibrosis was notably decreased.

The AFB1 + BSFL360 group demonstrated a marked reduction in collagen accumulation, with only sparse collagen fibers observed. The renal interstitium appeared well preserved, and glomerular architecture was largely restored, suggesting reduced collagen deposition and attenuated fibrotic remodeling at this dose.

#### 4.5.3. Quantitative Histopathological Analysis

Morphometric evaluation of renal injury parameters, including tubular necrosis (Figure 5C), necrotic glomeruli (Figure 5D), and interstitial inflammatory infiltration (Figure 5E), revealed significantly higher scores in the AFB1 group for all parameters than in the control group (P < 0.001 for all parameters). Treatment with BSFL extract dose-dependently reduced these scores, with the 360 mg/kg dose demonstrating the most substantial improvement (P < 0.05 to P < 0.001 vs. AFB1 group). The extract-alone group showed scores comparable to those of the control group, further confirming its safety.

Collectively, these histological and morphometric findings provide compelling evidence of the dose-dependent renoprotective effect of BSFL extract against AFB1-induced kidney injury, corroborating the biochemical and molecular data presented earlier.

## 5. Discussion

The pervasive contamination of food supplies with aflatoxin B1 continues to represent a major global public health concern, and its hepatotoxic consequences have been extensively documented over decades of research. Nevertheless, the nephrotoxic effects of AFB1 exposure have remained comparatively underexplored, which is particularly concerning given the kidney’s central role in concentrating and eliminating this toxin from the systemic circulation (1, 2). The present investigation provides, to the best of our knowledge, the first mechanistic characterization of how an n-hexane extract derived from black soldier fly larvae (*Hermetia illucens*) can mitigate AFB1-induced renal injury. Our experimental findings collectively indicate that this insect-derived lipid preparation acts through multiple interconnected protective mechanisms: it attenuates oxidative damage, curtails inflammatory signaling, readjusts the apoptotic balance, and preserves renal tissue integrity, with the Nrf2-mediated antioxidant response and the BAX/Bcl-2 apoptotic rheostat emerging as principal molecular pathways through which this protection is achieved (3, 4).

The pathophysiological cascade underlying AFB1 nephrotoxicity begins with the toxin’s metabolic conversion within renal proximal tubular cells. Following glomerular filtration, these epithelial cells take up AFB1 and subject it to biotransformation, primarily through cytochrome P450 enzymes, notably CYP3A4 and CYP1A2, generating the highly reactive electrophilic intermediate AFB1 - 8,9-epoxide (3, 5). This unstable metabolite readily forms covalent adducts with guanine residues in DNA, creating AFB1-N7-guanine lesions that not only have mutagenic potential but also initiate broader cellular stress responses (9). A particularly consequential outcome of this metabolic activation is the excessive generation of reactive oxygen species, including superoxide anion, hydrogen peroxide, and the highly reactive hydroxyl radical. These species inflict damage through several parallel routes: they peroxidize the polyunsaturated fatty acid components of membrane phospholipids—a process reflected in our observed elevation of malondialdehyde—they oxidize critical thiol groups on proteins, thereby inactivating essential antioxidant enzymes, including superoxide dismutase and catalase, and they deplete the intracellular glutathione reservoir that normally functions as a primary defense against electrophilic attack (5, 6).

The biochemical profile observed in our AFB1-exposed animals closely conforms to this established paradigm. We documented a pronounced elevation in renal MDA content alongside significant reductions in SOD and CAT activities, as well as marked GSH depletion. This pattern of redox disruption aligns with previous reports in rodent models of aflatoxin intoxication (4, 5) and underscores the particular vulnerability of renal tissue to oxidative challenge. This susceptibility arises from the kidney’s high metabolic rate, abundant oxidizable lipid content, and relatively modest complement of endogenous antioxidant defenses compared with hepatic tissue. The functional consequences of this oxidative injury were clearly reflected in the concurrent elevation of serum biomarkers indicating impaired renal filtration and reabsorptive function.

In parallel with the oxidative assault, AFB1 provokes a robust inflammatory response largely orchestrated by the transcription factor NF-κB. Under resting conditions, this factor remains sequestered in the cytoplasm through its association with the inhibitory protein IκB. However, oxidative stress induced by AFB1 activates the IκB kinase complex, which phosphorylates IκB and targets it for proteasomal degradation, thereby releasing NF-κB for nuclear translocation (7). Once in the nucleus, NF-κB drives the transcriptional activation of genes encoding pro-inflammatory mediators, including TNF-α, IL-1β, and IL-6, which collectively recruit immune cells, amplify chemokine production, and perpetuate tissue destruction (7). Our observation of significantly elevated TNF-α, IL-6, and NF-κB protein levels in the renal tissue of AFB1-treated animals provides direct evidence for engagement of this inflammatory cascade. Notably, this inflammatory response and the oxidative stress pathway are not independent but are mutually reinforcing: activated inflammatory cells generate additional ROS, which in turn sustain NF-κB activation, creating a self-perpetuating cycle of injury that can drive the transition from acute damage to chronic fibrotic remodeling.

Beyond these oxidative and inflammatory mechanisms, AFB1 disrupts the finely tuned regulation of mitochondrial apoptotic pathways, which is critically governed by the relative abundance of the pro-apoptotic effector BAX and its anti-apoptotic counterpart Bcl-2. The oxidative stress generated by AFB1 activates the tumor suppressor p53, which transcriptionally upregulates BAX expression while simultaneously repressing Bcl-2 (8). This shift in the BAX/Bcl-2 ratio favors permeabilization of the outer mitochondrial membrane, a pivotal event that releases cytochrome c into the cytosol and triggers the assembly of the apoptosome complex, culminating in caspase activation and irreversible cell death. Our Western blot analyses revealed a substantial increase in the BAX/Bcl-2 ratio in AFB1-exposed kidneys, consistent with ongoing apoptotic cell death within the tubular epithelium. This molecular alteration provides a mechanistic explanation for the concurrent elevation of serum BUN, creatinine, and uric acid levels, as the loss of functional tubular epithelial cells compromises the nephron’s capacity for filtration and solute reabsorption. The administration of n-hexane BSFL extract produced consistent, dose-dependent improvements across all parameters evaluated in this study. Although the extract’s complex lipid profile—dominated by lauric acid at 48% of total fatty acids, with additional contributions from myristic acid, palmitic acid, oleic acid, and minor components including tocopherols and sterols—likely underlies its pleiotropic protective effects, lauric acid may be a key contributor to the observed biological activity, based on previous reports demonstrating its protective effects in rodent models (8, 11). However, because we did not directly compare the whole extract with purified lauric acid or other isolated constituents, this interpretation remains a hypothesis requiring further investigation (8, 11). Of particular interest, the whole extract demonstrated superior efficacy compared with what would be predicted from the contribution of lauric acid alone—approximately 173 mg/kg at the higher dose—raising the possibility of synergistic interactions among the various lipid constituents (11, 12). However, because our study did not directly compare the whole extract with purified components, this synergistic effect remains a hypothesis that should be tested in future studies using bioassay-guided fractionation and direct comparison with isolated compounds (11, 12). This phenomenon of complex natural-product synergy is increasingly recognized as a distinguishing feature of whole extracts compared with isolated pure compounds.

Among the most notable findings from our molecular analyses was the restoration of Nrf2 expression in animals receiving the BSFL extract alongside AFB1. Treatment with BSFL extract reversed AFB1-induced Nrf2 suppression in a dose-responsive manner, and this recovery of Nrf2 expression correlated closely with the restoration of SOD, CAT, and GSH activities (9). Although these correlative data are consistent with Nrf2 pathway engagement, they do not establish causality. Nrf2 functions as a master regulator of antioxidant gene expression, and its activation typically requires nuclear translocation and binding to antioxidant response elements (AREs) in target gene promoters (9). Future studies employing selective Nrf2 inhibition, assessment of nuclear Nrf2 localization, or analysis of downstream target genes (HO-1, NQO1) will be necessary to confirm the mechanistic role of Nrf2 in the observed protective effect (9). This pattern is consistent with previous reports indicating that lauric acid and its metabolic derivative monolaurin can promote Nrf2 nuclear translocation, possibly through modification of Keap1 thiol groups or through engagement of upstream signaling cascades such as PI3K/Akt and MAPK pathways. Furthermore, the tocopherols present in the extract may contribute Nrf2-independent antioxidant protection through direct radical scavenging and membrane stabilization, thereby reducing the oxidative burden that the Nrf2 system would otherwise need to manage (11). This dual mechanism—indirect activation of endogenous antioxidant defenses combined with direct neutralization of reactive species—may explain the superior efficacy of the whole extract compared with lauric acid alone.

The anti-inflammatory action of the BSFL extract was clearly evidenced by significant reductions in TNF-α, IL-6, and NF-κB levels in treated groups. Because NF-κB serves as a central integrator of signals from diverse inflammatory pathways, its suppression indicates a broad rather than narrow anti-inflammatory effect. This attenuation may partly represent a secondary consequence of the extract’s antioxidant activity, given that ROS are potent drivers of IKK activation and subsequent IκB degradation (7). However, it is equally plausible that lauric acid or other lipid components act more directly on IKK or on upstream pattern recognition receptors such as Toll-like receptor 4, as has been demonstrated for other medium-chain fatty acids. This anti-inflammatory effect carries considerable clinical significance because TNF-α and IL-6 do more than sustain local inflammation; they also promote apoptosis and fibrosis, with TNF-α being particularly well established as an inducer of the pro-fibrotic cytokine TGF-β (7). By attenuating this inflammatory axis, the BSFL extract may help prevent the longer-term consequences of AFB1 exposure, including progressive interstitial fibrosis and eventual chronic kidney disease. The reduced collagen deposition observed on Masson’s trichrome staining in extract-treated animals provides suggestive histological evidence of attenuated fibrotic remodeling. However, because our assessment was primarily qualitative, the term “anti-fibrotic” should be interpreted with caution. Future studies using quantitative morphometric analysis of collagen-positive area and specific fibrosis markers (e.g., α-SMA, TGF-β1) are needed to confirm a true anti-fibrotic effect (23). Restoration of the BAX/Bcl-2 equilibrium represents another critical dimension of the extract’s protective action, as this ratio functions as a checkpoint for the mitochondrial apoptotic pathway. In our study, the elevated BAX/Bcl-2 ratio induced by AFB1 was substantially corrected by BSFL treatment, likely through several synergistic mechanisms. First, attenuation of oxidative stress would limit damage to mitochondrial DNA and lipids, preserving membrane integrity and preventing the release of pro-apoptotic factors (8). Second, Nrf2 activation itself can transcriptionally support Bcl-2 expression through downstream ARE-driven genes. Third, lauric acid has been reported to directly stabilize mitochondrial membranes, thereby limiting outer membrane permeabilization and cytochrome c release. The parallel increase in Bcl-2 and decrease in BAX observed in our study shifts the intracellular balance back toward cell survival, a conclusion strongly supported by the marked reduction in tubular necrosis seen in histopathological sections from extract-treated animals. This anti-apoptotic effect provides a mechanistic link between the molecular improvements and the preservation of functional renal architecture.

The histological findings reinforce and extend the biochemical and molecular data. AFB1-exposed kidneys displayed extensive tubular necrosis, pronounced interstitial inflammation, and substantial collagen deposition on Masson’s trichrome staining—features indicative of a transition from acute injury toward fibrotic remodeling. The BSFL extract, particularly at the higher dose, effectively curtailed these pathological changes. This anti-fibrotic effect is plausibly mediated through multiple mechanisms, including reduction of TGF-β1 (a downstream product of NF-κB/TNF-α signaling that drives fibroblast activation and collagen synthesis) and limitation of epithelial-to-mesenchymal transition, a process promoted by both oxidative stress and inflammation. By restraining these upstream drivers, the extract may interrupt progression toward fibrosis and help preserve long-term renal architecture and function. The extract-alone group exhibited histology comparable to that of healthy controls, confirming the absence of intrinsic toxicity at the tested dose.

When considered alongside previous reports on isolated lauric acid or other plant-derived phytochemicals, the BSFL extract appears to offer a broader spectrum of protective effects, most likely because it delivers a complex lipid mixture rather than a single compound. Isolated lauric acid has been shown to provide hepatoprotection at doses of 100 - 250 mg/kg; the 360 mg/kg BSFL dose used here supplied approximately 173 mg/kg of lauric acid, which falls within this effective range. Nevertheless, the whole extract performed better than would be predicted from lauric acid’s projected contribution alone, suggesting that synergy among its various fatty acids and minor lipid constituents—such as oleic acid, palmitoleic acid, and tocopherols—may enhance lauric acid’s bioavailability and membrane-stabilizing action. Compared with other natural products previously investigated for AFB1 nephroprotection, such as Gallic acid (4) and luteolin (5), the BSFL extract offers distinct advantages in terms of sustainability and scalability of production.

Beyond its pharmacological relevance, the use of black soldier fly larvae as a lipid source fits within a broader circular economy framework. *H. illucens* larvae efficiently convert low-value organic residues—food waste, agricultural by-products, olive pomace—into biomass rich in protein and lipids (10, 13). In this study, the larvae were reared specifically on fruit and olive processing residues, diverting material that would otherwise contribute to environmental pollution. Because the larvae grow rapidly and can be reared at scale with comparatively minimal land, water, and energy inputs, this system is well suited to industrial production (11), and it aligns with UN Sustainable Development Goals 12 and 13. Unlike palm or coconut oil, BSFL oil production does not compete with food crops for arable land, thereby circumventing the deforestation and biodiversity loss associated with conventional plant oil agriculture. Its fatty acid profile, dominated by medium-chain saturated fatty acids, could also reduce dependence on imported plant oils in food and pharmaceutical applications (11). In this sense, the present findings support both a therapeutic rationale and a broader ecological and economic case for insect-derived oils.

**Figure 6.**
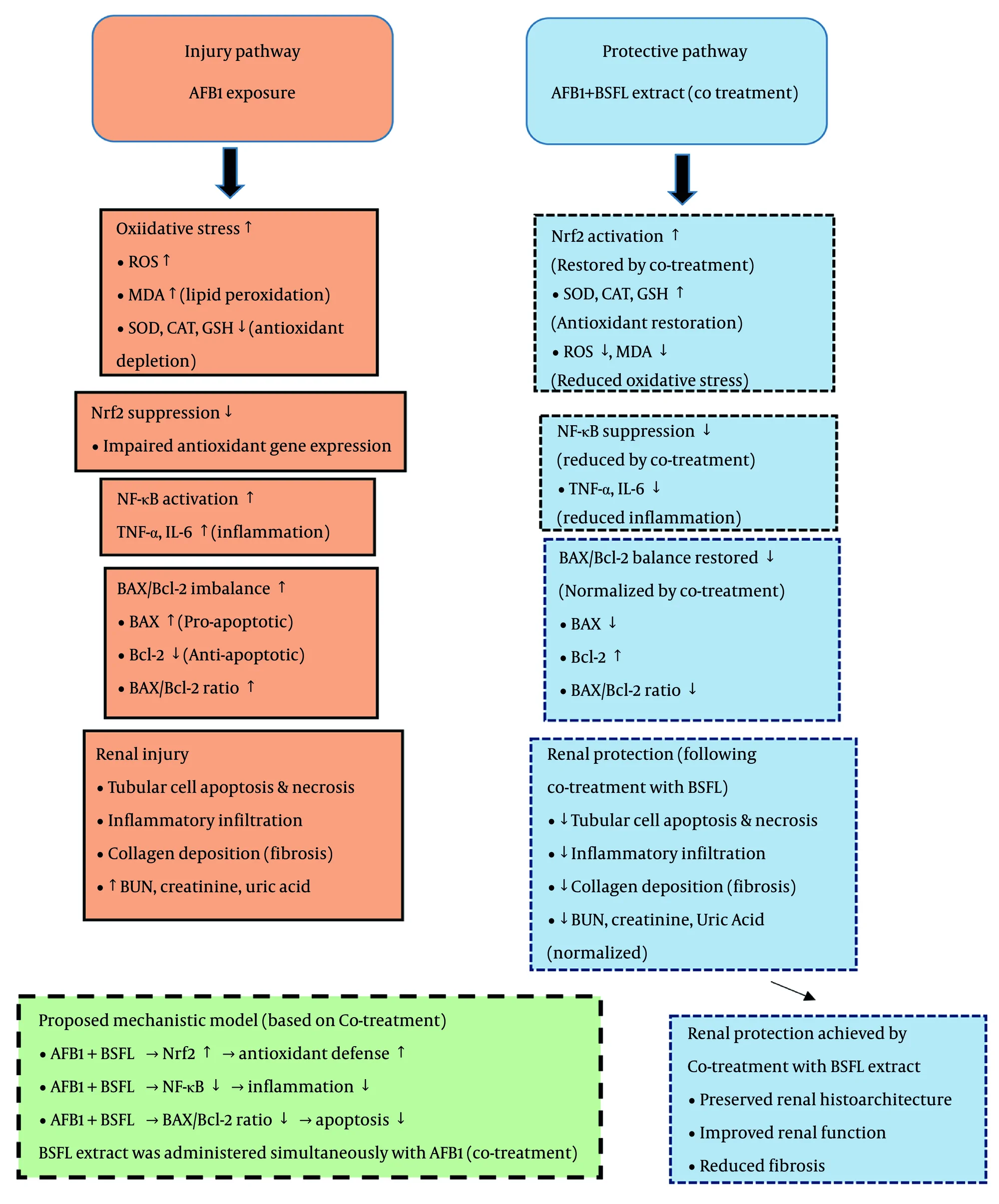
Proposed mechanistic model of BSFL extract-mediated renoprotection against AFB1-induced kidney injury. The figure illustrates two parallel pathways. Injury pathway (left): AFB1 exposure drives oxidative stress (↑ MDA; ↓ SOD, CAT, GSH), NF-κB-mediated inflammation (↑ TNF-α, IL-6), and BAX/Bcl-2 imbalance (↑ BAX/Bcl-2 ratio), leading to tubular necrosis, fibrosis, and renal dysfunction. Protective pathway (right): Co-treatment with BSFL extract counteracts these effects by restoring Nrf2 expression, suppressing NF-κB, and normalizing the BAX/Bcl-2 ratio, thereby preserving renal function and histoarchitecture. Solid lines: established effects supported by previous literature; dashed lines: pathways inferred from correlative data obtained in this study, suggesting but not proving mechanistic involvement. Blue arrows: protective effects of BSFL extract; orange arrows: injurious effects of AFB1. This model is proposed as a working hypothesis requiring further experimental validation.

Based on the correlative evidence presented above, we propose a mechanistic model (Figure 6) in which BSFL extract exerts its renoprotective effects through coordinated modulation of three interconnected pathways: Nrf2-mediated antioxidant defense (9), NF-κB-driven inflammatory signaling (7), and BAX/Bcl-2-regulated apoptotic balance (8, 9). In this model, AFB1 generates ROS that simultaneously suppress Nrf2, activate NF-κB, and shift the BAX/Bcl-2 ratio toward cell death (1-6); BSFL extract, through its lipid constituents (particularly lauric acid and potentially other bioactive components) (8, 11, 12), counteracts these effects by restoring Nrf2 expression, limiting NF-κB activation, and normalizing apoptotic balance. The net result is preservation of renal function, reduction of tissue injury, and attenuation of fibrotic remodeling (4-6) (Figure 6). In this model, solid lines represent well-established effects of AFB1 and BSFL extract supported by previous literature (1-9), whereas dashed lines indicate pathways inferred from the correlative data obtained in the present study, which suggest—but do not definitively prove—mechanistic involvement (9). However, we emphasize that this model is proposed based on correlative data and should be regarded as a working hypothesis requiring experimental validation through pathway-specific inhibition, nuclear translocation studies, and analysis of downstream target genes (9).

Several limitations of the present study warrant acknowledgment. First, we tested a crude extract rather than isolated compounds; although lauric acid is a major constituent (48% of total fatty acids), we did not compare the whole extract with purified lauric acid or other individual components. Whether the protective effect is attributable primarily to lauric acid, to other constituents, or to synergistic interactions requires future investigation through bioassay-guided fractionation (8, 11, 12). Second, our model used short-term, high-dose AFB1 exposure, which does not fully recapitulate chronic, low-dose dietary exposure in humans; chronic studies are needed to assess long-term protection (4). Third, our mechanistic evidence is primarily correlative and based on protein expression data. Although the coordinated changes in Nrf2, NF-κB, and BAX/Bcl-2 suggest pathway involvement, our data demonstrate association rather than causality. Future studies using selective inhibitors (e.g., ML385), assessment of nuclear translocation, or gene silencing approaches are required to establish causal relationships (9). Fourth, the extract appeared safe over 28 days, but comprehensive toxicological characterization would be required before human application. Fifth, our histopathological assessment of collagen deposition via Masson’s trichrome staining, although qualitatively analyzed, indicated reduced collagen deposition. However, the term 'anti-fibrotic' should be interpreted with caution, and future studies using specific fibrosis markers (e.g., α-SMA, TGF-β1, collagen I/III) and longer treatment durations are needed to confirm a true anti-fibrotic effect (23). Sixth, this study used only male rats; future research should include females to assess potential sex differences, given that estrogenic and androgenic pathways can modulate oxidative stress and Nrf2 signaling.

### 5.1. Conclusions

Taken together, n-hexane oil from black soldier fly larvae affords substantial protection against AFB1-induced kidney injury, in association with coordinated modulation of Nrf2-driven antioxidant defenses, NF-κB-mediated inflammatory responses, and BAX/Bcl-2 apoptotic signaling. This multi-targeted action translated into preserved renal function, reduced tissue damage, and attenuated fibrosis in our rat model. Although lauric acid appears to be a key active constituent, synergistic contributions from other fatty acids and minor lipid components are likely. Beyond its biological efficacy, the sustainable and environmentally friendly production of BSFL oil positions it as a promising natural therapeutic candidate for mitigating mycotoxin-associated nephrotoxicity. These findings provide a strong foundation for future research, including the isolation of specific bioactive compounds, elucidation of their precise molecular targets, and translation to relevant chronic models of kidney injury.

## Data Availability

The dataset presented in the study is available on request from the corresponding author during submission or after publication.
